# Immune cell metabolic reprogramming in pancreatic cancer: roles in tumor progression and therapeutic strategies

**DOI:** 10.3389/fimmu.2026.1968254

**Published:** 2026-10-29

**Authors:** Gangzhao Gu, Yang Du, Hengzhi Zhang, Yu Zheng, Yanjun Yao, Qiang Yan

**Affiliations:** 1 Department of Hepatobiliary and Pancreatic Surgery, Huzhou University Affiliated Central Hospital, Huzhou, Zhejiang, China; 2 Department of Hepatobiliary and Pancreatic Surgery, Huzhou Hospital Affiliated to Zhejiang University, Huzhou, Zhejiang, China; 3 Department of Hepatobiliary and Pancreatic Surgery, Huzhou Central Hospital, Fifth School of Clinical Medicine of Zhejiang Chinese Medical University, Huzhou, Zhejiang, China; 4 Department of Hepatobiliary and Pancreatic Surgery, Lishui Central Hospital, Lishui, Zhejiang, China

**Keywords:** immune cells, immunotherapy, metabolic reprogramming, metabolism-targeted therapy, pancreatic cancer, tumor immune microenvironment

## Abstract

Pancreatic cancer is one of the most lethal malignancies of the digestive system, and its profoundly immunosuppressive tumor microenvironment (TME) represents a major driver of tumor progression and therapeutic resistance. Emerging evidence indicates that metabolic reprogramming profoundly influences the polarization, effector function, survival, and fate of immune cells within the TME, thereby shaping the balance between antitumor immunity and immune escape. However, substantial challenges remain, including the incompletely understood metabolic heterogeneity among distinct immune cell subsets and the limited clinical translation of therapeutic strategies combining metabolic intervention with immunotherapy. In this review, we summarize the metabolic reprogramming characteristics and underlying molecular mechanisms of major immune cell populations in pancreatic cancer, including T cells, tumor-associated macrophages (TAMs), myeloid-derived suppressor cells (MDSCs), and natural killer (NK) cells, with particular emphasis on their reciprocal interactions with pancreatic cancer progression and the immunosuppressive microenvironment. We further discuss recent advances in metabolism-targeted therapies and immunotherapy, highlighting the therapeutic potential of combination strategies centered on immunometabolic modulation. Finally, we outline current challenges and emerging opportunities in this rapidly evolving field, with the aim of providing a mechanistic framework and translational perspectives for the development of precision therapeutic strategies for pancreatic cancer.

## 1 Introduction

Pancreatic cancer is a highly aggressive malignancy characterized by late diagnosis, rapid progression, and poor therapeutic responsiveness. Although advances have been made in surgery, chemotherapy, and radiotherapy, clinical outcomes remain unsatisfactory, while immune checkpoint inhibitors have shown limited efficacy. This resistance is largely attributable to the unique TME, which is characterized by extensive desmoplasia, hypoxia, nutrient deprivation, and profound immunosuppression (1). Activated pancreatic stellate cells and cancer-associated fibroblasts (CAFs) generate a dense extracellular matrix (ECM) that restricts immune-cell infiltration and promotes the accumulation of regulatory T cells (Tregs), M2-like macrophages, and MDSCs while reducing CD8^+^ T-cell, CD4^+^ T-cell, and NK-cell infiltration (2). Collagen-rich stroma further impairs effective T-cell migration toward tumor cells (3). Hypoxia enhances extracellular adenosine accumulation through HIF-dependent pathways, suppressing T cells, NK cells, and dendritic cells while promoting immunosuppressive macrophage polarization (4). Together, these factors contribute to the “cold tumor” phenotype of pancreatic cancer, characterized by sparse and frequently exhausted cytotoxic T-cell infiltration (5).

Metabolic reprogramming has emerged as a critical mechanism linking the hostile pancreatic cancer microenvironment to immune dysfunction. TAMs undergo lipid-dependent metabolic adaptation, including increased CD36-mediated lipid uptake and fatty acid oxidation (FAO), thereby acquiring immunosuppressive phenotypes that inhibit CD8^+^ T and NK cells (6). Tumor-intrinsic DGAT1 reshapes lipid availability and promotes CD8^+^ T-cell dysfunction, whereas DGAT1 inhibition preserves FOXO1 activity and supports stem-like T-cell differentiation (7). Polyamine metabolism driven by the KRAS–MYC axis promotes M2-like macrophage polarization, MDSC expansion, and T-cell exhaustion (8). Therapeutically, PTK6 links altered lipid metabolism with ferroptosis resistance and T-cell exhaustion (9), whereas PI3Kδ inhibition can remodel both fibrotic and immunosuppressive components of the pancreatic cancer microenvironment (10). In addition, copper-delivery nanoplatforms combined with irreversible electroporation can induce cuproptosis while enhancing dendritic-cell maturation, CD8^+^ T-cell infiltration, and M1 macrophage polarization (11). These findings highlight immune metabolic reprogramming as a promising target for overcoming therapeutic resistance in pancreatic cancer.

## 2 Metabolic characteristics of the pancreatic cancer immune microenvironment

### 2.1 Metabolic ecosystem of the pancreatic cancer microenvironment

The TME of pancreatic cancer is characterized by a highly desmoplastic stroma, which is composed of abundant activated pancreatic stellate cells, CAFs, and ECM components, collectively forming both physical and metabolic barriers (12). These stromal cells are highly metabolically active and compete with tumor cells and infiltrating immune cells for limited nutrients, including glucose and glutamine, thereby contributing to profound nutrient deprivation within the tumor milieu (13). At the same time, pancreatic cancer cells exhibit pronounced aerobic glycolysis through the Warburg effect, resulting in excessive lactate production and accumulation and, consequently, the establishment of an acidic microenvironment. Such metabolic conditions not only directly impair the cytotoxic activity of CD8^+^ T cells and NK cells but also favor the expansion of Tregs and the polarization of TAMs toward an immunosuppressive M2-like phenotype, thereby reinforcing an immunosuppressive metabolic niche (14, 15).

Hypoxia is another prominent metabolic feature of the pancreatic cancer microenvironment. Stabilization of hypoxia-inducible factor-1α (HIF-1α) induces extensive metabolic remodeling by upregulating glycolysis-related enzymes and transporters, including glucose transporter 1 (GLUT1) and lactate dehydrogenase A (LDHA), further intensifying metabolic stress and promoting adaptive metabolic changes in infiltrating immune cells (16). Lipid metabolism is also markedly dysregulated in pancreatic cancer, with enhanced fatty acid synthesis, uptake, storage, and β-oxidation. These lipid metabolic alterations not only provide energy and biosynthetic substrates for malignant cells but also generate bioactive lipid mediators that regulate immune-cell differentiation and function, thereby establishing a complex metabolic–immune crosstalk network (17). Importantly, pancreatic cancer cells and stromal cells engage in extensive metabolic symbiosis. CAFs can provide metabolic substrates and intermediates that support tumor-cell survival and proliferation under nutrient-deprived conditions, whereas tumor-derived soluble factors reciprocally reprogram stromal-cell metabolism. This bidirectional metabolic interaction further reinforces the unique metabolic ecosystem of pancreatic cancer and contributes to the maintenance of tumor growth, immune suppression, and therapeutic resistance (18).

### 2.2 Key pathways of immune cell metabolic reprogramming

Immune cell metabolic reprogramming refers to the dynamic remodeling of cellular metabolic pathways in response to environmental and signaling cues, allowing immune cells to transition between quiescent, activated, differentiated, and dysfunctional states. This process involves coordinated changes in glycolysis, oxidative phosphorylation (OXPHOS), FAO, amino acid metabolism, and the pentose phosphate pathway (19, 20). Rather than merely supplying energy, these metabolic programs provide biosynthetic precursors and signaling intermediates that directly influence immune-cell differentiation and function. In T cells, metabolic fate is tightly coupled to functional differentiation. Activated effector T cells rely predominantly on aerobic glycolysis and glutamine metabolism to support rapid proliferation, cytokine production, and biosynthesis, whereas Tregs generally exhibit greater dependence on mitochondrial oxidative metabolism and FAO to sustain survival and suppressive activity under nutrient-limited conditions (21). In pancreatic cancer, this metabolic adaptability may contribute to the persistence of suppressive T-cell states within glucose-poor and hypoxic tumor regions.

Macrophage polarization is likewise coupled to extensive metabolic remodeling. M1-like programs are typically associated with enhanced glycolytic flux and inducible nitric oxide synthase (iNOS)-dependent arginine metabolism, whereas M2-like programs are linked to mitochondrial respiration, lipid utilization, and arginase-1 (ARG1)-mediated ornithine production (22). In pancreatic cancer, these canonical programs are further reshaped by hypoxia, lipid availability, glutamine metabolism, and tumor-derived metabolites, generating metabolically diverse macrophage states with distinct immunoregulatory functions. The PI3K/AKT/mTOR and AMP-activated protein kinase (AMPK) pathways, together with transcription factors such as c-Myc and HIF-1α, constitute major regulatory hubs linking extracellular cues to metabolic reprogramming (23). Aberrant PI3K/AKT/mTOR signaling is frequently observed in pancreatic cancer and contributes not only to tumor-cell metabolic adaptation but also to the establishment of an immunosuppressive microenvironment by altering the metabolic states of surrounding immune cells (24). Metabolic regulation is also closely integrated with epigenetic control. Several metabolic intermediates, including acetyl-CoA, α-ketoglutarate, and S-adenosylmethionine, act as substrates or cofactors for chromatin-modifying enzymes. Changes in the abundance of these metabolites can therefore influence histone acetylation, histone and DNA methylation, and transcriptional programs governing immune-cell activation, differentiation, and exhaustion (25). This metabolic–epigenetic coupling provides a mechanistic explanation for how transient environmental metabolic stress can be translated into relatively stable immune-cell phenotypes.

### 2.3 Metabolic reprogramming as a determinant of immune cell function and fate

Metabolic reprogramming profoundly influences immune-cell activation thresholds, proliferative capacity, cytokine production, cytotoxic activity, survival, and differentiation, thereby determining both the magnitude and durability of antitumor immune responses (26). In the pancreatic cancer microenvironment, competition for nutrients between tumor cells and immune cells represents an important mechanism of immune escape. Effector T cells exposed to glucose depletion, amino acid restriction, hypoxia, and metabolite accumulation progressively lose metabolic fitness, resulting in impaired proliferation, reduced cytokine production, defective cytotoxicity, and eventual functional exhaustion. By contrast, immunosuppressive populations such as Tregs and MDSCs often display greater metabolic adaptability, allowing them to retain or even enhance their suppressive functions under nutrient-poor and hypoxic conditions (27). This differential metabolic fitness contributes to a self-reinforcing immunosuppressive ecosystem.

Metabolites themselves can also function as signaling molecules and epigenetic regulators rather than merely serving as metabolic substrates. Lactate is a representative example. In addition to altering cellular redox balance and extracellular acidity, lactate can regulate immune-cell function through HIF-dependent signaling and protein lactylation (28). In pancreatic cancer, elevated tumor-derived lactate has been reported to promote ENSA K63-linked lactylation and activate the signal transducer and activator of transcription 3 (STAT3)/CCL2 signaling axis, thereby enhancing TAM recruitment and the establishment of an immunosuppressive phenotype. These changes ultimately contribute to remodeling of the tumor immune microenvironment and resistance to immunotherapy (29). Another important concept is metabolic memory, whereby transient metabolic exposure can induce durable changes in immune-cell functional programs through sustained transcriptional and epigenetic remodeling. Such metabolic memory may contribute to both trained immunity and immune tolerance, thereby influencing how immune cells respond to subsequent stimuli (30). This phenomenon also provides a potential therapeutic window for reprogramming dysfunctional immune cells rather than simply inhibiting individual metabolic enzymes.

Recent studies further suggest that metabolic checkpoints can be therapeutically manipulated to restore immune-cell fitness. For example, targeting uncoupling protein 2 (UCP2) has been reported to reprogram CD8^+^ T-cell metabolism, enhance mitochondrial OXPHOS, and increase interferon-γ (IFN-γ) production, thereby restoring antitumor immune activity in pancreatic cancer (31). Collectively, these findings indicate that immune-cell metabolism is not merely a consequence of the pancreatic cancer microenvironment but a central determinant of immune-cell fate and function. Understanding these metabolic programs will therefore be essential for developing strategies capable of reversing immune dysfunction and improving therapeutic responses in pancreatic cancer.

## 3 Metabolic reprogramming of immune cells in pancreatic cancer

### 3.1 Metabolic exhaustion and functional dysfunction of CD8^+^ T cells

Within the pancreatic cancer microenvironment, persistent antigen exposure and chronic T-cell receptor (TCR) stimulation progressively couple inhibitory checkpoint signaling to metabolic failure in CD8^+^ T cells. Sustained engagement of PD-1 and TIM-3 attenuates PI3K–AKT–mTOR activity, limiting GLUT1-dependent glucose uptake, glycolytic flux, and anabolic metabolism required for proliferation and effector function (32). As nutrient availability declines, T cells increasingly fail to maintain the metabolic flexibility required to support sustained cytokine production and cytotoxicity. Reduced mTOR activity also compromises mitochondrial biogenesis and respiratory capacity, thereby narrowing the bioenergetic reserve available during prolonged activation.

Mitochondrial dysfunction subsequently becomes a central amplifier of T-cell exhaustion. Persistent stimulation promotes mitochondrial fragmentation, defective mitophagy, and impaired turnover of damaged organelles, leading to accumulation of dysfunctional mitochondria and excessive reactive oxygen species (ROS) (33). This process disrupts NAD^+^/NADH balance, constrains redox buffering, and further limits oxidative metabolism, creating a self-reinforcing cycle of bioenergetic stress and oxidative damage (34). The lactate-rich and acidic pancreatic cancer microenvironment intensifies this metabolic pressure by restricting lactate export, perturbing intracellular redox homeostasis, and suppressing glycolytic activity. These metabolic constraints reinforce the exhausted phenotype and limit sustained antitumor immunity. Several metabolic checkpoints have emerged as potential therapeutic targets for restoring CD8^+^ T-cell function (35). Amino-acid availability and redox metabolism further shape CD8^+^ T-cell fitness within pancreatic cancer. Serine biosynthesis, glutamine utilization, and glutathione-dependent antioxidant capacity influence nucleotide synthesis, mitochondrial function, and redox balance, thereby affecting proliferative and effector competence under nutrient restriction (36). A deeper understanding of the metabolic determinants of CD8^+^ T-cell dysfunction may provide a mechanistic basis for developing strategies to improve the efficacy of immunotherapy in pancreatic cancer.

### 3.2 Metabolic polarization of TAMs and their immunosuppressive functions

TAMs constitute a dominant immunosuppressive population in pancreatic cancer and undergo extensive metabolic adaptation characterized by altered lipid handling, mitochondrial respiration, glutamine utilization, and arginine metabolism (37). Enhanced fatty acid uptake and oxidative metabolism can sustain macrophage survival and immunoregulatory activity under nutrient limitation, while ARG1-dependent arginine catabolism supports polyamine and proline synthesis, thereby linking macrophage metabolism to tissue remodeling, angiogenesis, and tumor progression (38). Thus, altered arginine metabolism contributes both directly and indirectly to tumor progression. TAM metabolism remains highly plastic across tumor evolution. Glycolysis, pentose phosphate pathway activity, lipid-droplet accumulation, and PPARγ-associated transcriptional programs can coexist within tumor-supportive macrophage states (39, 40). Importantly, macrophage metabolic phenotypes are highly plastic and can change dynamically during tumor evolution. Early inflammatory macrophage programs may gradually transition toward immunosuppressive, tissue-remodeling states as pancreatic cancer progresses (41).

CD36-dependent lipid metabolism reshapes the immunoregulatory output of TAMs. Enhanced lipid utilization is associated with increased production of IL-10 and transforming growth factor-β (TGF-β), reinforcing local immune suppression and stromal remodeling (42). In parallel, altered lipid metabolism can modify phagocytic activity and the handling of dying tumor cells, thereby affecting the inflammatory signals generated after engulfment. Through IL-10, TGF-β, nutrient competition, and lipid-derived mediators, metabolically adapted TAMs suppress CD8^+^ T-cell activation and cytotoxicity and diminish NK-cell effector activity, creating a lipid-dependent immunosuppressive circuit within pancreatic cancer (43). This temporal metabolic plasticity provides tumor cells with a progressively more favorable immune niche and may contribute to the development of therapeutic resistance. Consequently, targeting macrophage metabolism to redirect TAMs from tumor-promoting toward antitumor states represents an attractive strategy for remodeling the pancreatic cancer immune microenvironment.

### 3.3 Metabolic adaptation, expansion, and enhanced suppressive activity of MDSCs

MDSCs are markedly enriched in pancreatic cancer and display pronounced metabolic plasticity that supports their persistence within a nutrient-poor and inflammatory tumor milieu. Glycolysis, mitochondrial oxidative metabolism, and FAO can operate in parallel, allowing these cells to maintain suppressive activity despite fluctuating nutrient and oxygen availability (44). This metabolic plasticity is critical for their accumulation and persistence within pancreatic tumors. A major mechanism by which MDSCs suppress antitumor immunity is the depletion of amino acids required for T-cell activation and proliferation. Through high expression of ARG1 and iNOS, MDSCs extensively metabolize arginine and disrupt its availability to effector T cells (45). MDSCs can also interfere with the metabolism of tryptophan and cysteine, further imposing nutrient stress on T cells and inducing cell-cycle arrest, impaired protein synthesis, and functional anergy. These nutrient-deprivation mechanisms establish a metabolically restrictive microenvironment that selectively disadvantages antitumor lymphocytes.

In addition, MDSCs generate large amounts of ROS and reactive nitrogen species, which contribute directly to T-cell dysfunction. These reactive intermediates can modify components of the TCR-CD3 complex, interfere with antigen recognition, induce apoptosis, and alter chemokine gradients through nitration, thereby reducing T-cell trafficking into tumor tissues (46, 47). The transcription factors STAT3 and CCAAT/enhancer-binding protein-β (C/EBPβ) are central regulators of MDSC expansion and suppressive programming. Sustained activation of these pathways promotes abnormal myeloid differentiation and supports metabolic adaptations that further reinforce the immunosuppressive phenotype (48). Lipid metabolism is particularly important for the maintenance of MDSC function. Increased uptake of long-chain fatty acids and enhanced FAO provide ATP and preserve mitochondrial integrity, thereby promoting MDSC survival and suppressive activity under nutrient-stressed conditions (49). These metabolic adaptations make MDSCs a key component of the immunosuppressive network in pancreatic cancer and an important barrier to effective immunotherapy.

### 3.4 Metabolic characteristics of NK cells and Tregs

NK cells in the pancreatic cancer microenvironment often exhibit impaired cytotoxic activity accompanied by pronounced metabolic dysfunction. Both glycolytic capacity and mitochondrial respiration can be suppressed, leading to reduced IFN-γ production, defective degranulation, and diminished tumor-cell killing (50, 51). Hypoxia and nutrient deprivation inhibit mechanistic target of rapamycin (mTOR) signaling in NK cells, limiting the metabolic activation required for optimal effector function. In parallel, lactate accumulation and extracellular acidosis can further impair NK-cell activity by interfering with cellular metabolism and reducing the expression or function of activating receptors. Following activating-receptor and cytokine stimulation, mTORC1 and c-Myc function as major metabolic regulators that increase glucose uptake, glycolytic flux, amino-acid utilization, and biosynthetic activity while supporting mitochondrial respiration. This metabolic transition provides the ATP and biosynthetic intermediates required for granzyme and perforin production, degranulation, and IFN-γ secretion (52). TGF-β and adenosine provide additional layers of metabolic suppression. TGF-β restrains mTORC1-dependent metabolic activation and reduces the biosynthetic and mitochondrial programs required for effective NK-cell responses. These metabolic constraints collectively reduce NK-cell surveillance and contribute to immune escape in pancreatic cancer (53). In contrast, Tregs display substantial metabolic flexibility that favors their persistence within glucose-poor, hypoxic, and metabolically constrained tumor regions. Relative to highly glycolytic effector T cells, Tregs frequently engage mitochondrial OXPHOS and fatty acid utilization to sustain survival and suppressive function (54). This metabolic preference contributes to the selective persistence of Tregs in the pancreatic cancer microenvironment.

Tregs also express high levels of inhibitory and costimulatory molecules, including cytotoxic T-lymphocyte-associated protein 4 (CTLA-4) and glucocorticoid-induced tumor necrosis factor receptor-related protein (GITR), which support their suppressive activity and can interact with intracellular signaling networks that regulate metabolic adaptation (55). Furthermore, Tregs compete with effector T cells for essential growth and metabolic resources. Their high expression of the interleukin-2 (IL-2) receptor enables efficient consumption of IL-2, while competition for glucose and other nutrients can further restrict the metabolic activation and expansion of conventional T cells (56, 57). This unequal metabolic competition creates a selective advantage for immunosuppressive T-cell populations and reinforces the immune-resistant state of pancreatic cancer. Collectively, the distinct metabolic programs adopted by CD8^+^ T cells, TAMs, MDSCs, NK cells, and Tregs illustrate that immune-cell metabolism is highly heterogeneous within the pancreatic cancer microenvironment. Antitumor immune cells are frequently metabolically constrained and driven toward dysfunction, whereas immunosuppressive populations develop adaptive metabolic programs that sustain their survival and suppressive capacity (58). This imbalance constitutes a central component of pancreatic cancer immune evasion and provides a strong rationale for immune-cell-specific metabolic intervention (**Figure 1**).

**Figure 1:**
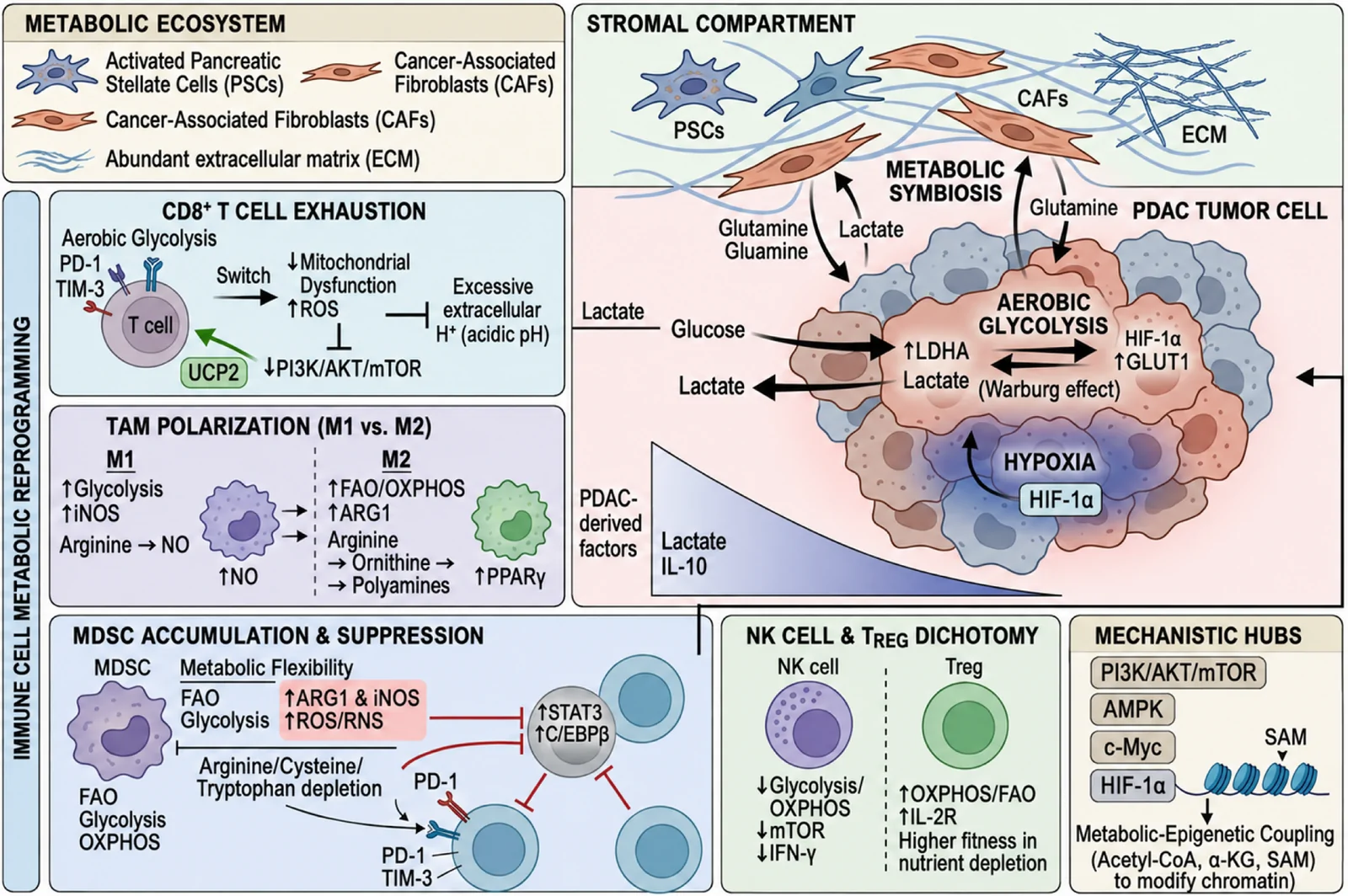
Metabolic reprogramming of the pancreatic cancer immune microenvironment.

## 4 Mechanisms of immune cell metabolic reprogramming in pancreatic cancer progression

### 4.1 Metabolite-mediated local immunosuppression and promotion of tumor invasion

Pancreatic cancer cells release abundant metabolic enzymes, lactate, and lipid-derived metabolites into the TME through extracellular vesicles and paracrine signaling, thereby establishing a metabolic ecosystem that favors tumor progression. These metabolites directly reshape the functional phenotypes of infiltrating immune cells and promote metabolic symbiosis between malignant and stromal or immune compartments. For example, tumor-derived lactate can enter immune cells through monocarboxylate transporters and alter intracellular pH and redox homeostasis, thereby suppressing nuclear factor of activated T cells (NFAT) activity and reducing IL-2 expression in T cells (59). Lactate can also enhance ARG1 expression in MDSCs, further strengthening the immunosuppressive network. In addition to lactate, metabolites such as kynurenine, adenosine, and prostaglandin E2 (PGE2) contribute substantially to immune suppression in pancreatic cancer. These metabolites activate the aryl hydrocarbon receptor (AhR), adenosine receptors such as ADORA2B, and prostaglandin E receptors on immune cells, respectively, thereby cooperatively suppressing the antitumor functions of T cells and NK cells while promoting the differentiation and accumulation of Tregs (60). Free fatty acids and oxidized cholesterol derivatives can further influence macrophage and myeloid-cell signaling through pattern-recognition pathways, including Toll-like receptors and the NLRP3 inflammasome, thereby altering inflammatory programs that may favor tumor invasion and progression (61). Metabolic reprogramming also affects ECM remodeling. By altering glucose and glutamine utilization, pancreatic cancer cells can influence the metabolic activity of stromal cells and thereby modulate matrix metalloproteinase activity, hyaluronan production, and ECM deposition (62). These changes facilitate stromal remodeling, vascular dysfunction, and tumor-cell invasion. Thus, metabolite accumulation in pancreatic cancer does not merely reflect altered tumor metabolism but actively participates in immune suppression, stromal remodeling, and malignant progression.

### 4.2 Immune metabolic reprogramming-driven angiogenesis and stromal remodeling

Metabolically reprogrammed TAMs are important regulators of angiogenesis and desmoplasia in pancreatic cancer. TAMs can upregulate vascular endothelial growth factor (VEGF), matrix metalloproteinase-9 (MMP-9), and platelet-derived growth factor (PDGF), thereby promoting abnormal neovascularization and stromal fibrosis (63). These processes worsen tissue hypoxia and further disrupt nutrient distribution within the tumor, creating conditions that reinforce metabolic stress and immune dysfunction. Under hypoxic conditions, stabilization of HIF-1α in macrophages enhances the expression of glycolysis-associated genes and can redirect arginine metabolism toward ornithine and polyamine biosynthesis (64). Polyamines, in turn, support endothelial-cell proliferation and migration and may therefore contribute to the establishment of a hypoxia–metabolism–angiogenesis feedback loop (65). This reciprocal relationship enables hypoxia to promote both immunosuppressive macrophage programming and vascular remodeling, while newly formed but structurally abnormal vessels fail to restore adequate perfusion.

MDSCs also contribute to stromal remodeling through signaling pathways closely linked to their metabolic state. Persistent activation of STAT3 can promote the production of growth factors and stromal-modulating mediators, including fibroblast growth factor and hepatocyte growth factor, which may participate in pancreatic stellate cell activation and excessive ECM deposition (66). Moreover, immune-cell-derived cytokines such as IL-17 and TGF-β can influence endothelial metabolism and promote endothelial-to-mesenchymal transition, thereby increasing vascular permeability and facilitating tumor-cell dissemination (67). Abnormal angiogenesis subsequently aggravates hypoperfusion and hypoxia, further enhancing glycolytic metabolism, lactate accumulation, and immune suppression (68). These reciprocal interactions create a self-reinforcing network in which immune-cell metabolic reprogramming, vascular dysfunction, and stromal remodeling mutually sustain one another and collectively promote pancreatic cancer progression.

### 4.3 Crosstalk between metabolic reprogramming and epigenetic regulation

Metabolic reprogramming can reshape immune-cell transcription through changes in chromatin accessibility and post-translational modification. Acetyl-CoA, α-ketoglutarate, and S-adenosylmethionine connect central carbon and one-carbon metabolism with histone acetylation, demethylation, and methylation, providing a biochemical route through which nutrient availability influences immune-cell state (69). In pancreatic cancer, this metabolic–epigenetic interface is particularly relevant because hypoxia, nutrient restriction, and high glycolytic flux continuously alter the local abundance of metabolites capable of modifying chromatin-regulatory activity. Lactate represents the most prominent link between altered tumor metabolism and epigenetic remodeling in pancreatic cancer. Beyond its effects on extracellular acidity and redox balance, lactate can drive lysine lactylation of histone and non-histone proteins, thereby altering inflammatory and immune-regulatory transcriptional programs (70). In pancreatic cancer, lactylation-associated signaling has been linked to macrophage recruitment, immunosuppressive remodeling, stromal adaptation, and therapeutic resistance. The ENSA lactylation/STAT3/CCL2 axis provides a mechanistic example in which tumor-derived lactate is translated into a transcriptional program that reinforces myeloid-cell accumulation and immune suppression. Lactylation also extends beyond macrophage regulation. Increased H3K18 lactylation has been associated with aggressive pancreatic cancer behavior, while lactylation of proteins involved in stromal remodeling and metastatic progression further connects glycolytic metabolism with tissue organization and tumor evolution (71). Together, these findings position lactate-dependent protein modification as a central metabolic–epigenetic mechanism linking malignant-cell metabolism to persistent immune and stromal reprogramming in pancreatic cancer.

### 4.4 Intercellular metabolic crosstalk and metabolic symbiosis networks

Pancreatic cancer cells and immune cells establish extensive metabolic crosstalk that contributes to the maintenance of an immunosuppressive microenvironment. Tumor-derived cytokines, extracellular vesicles, metabolic enzymes, and metabolites can reprogram immune-cell metabolism and promote the acquisition of tumor-supportive phenotypes (72). In turn, metabolically altered immune cells release nutrients, cytokines, and metabolites that can sustain malignant-cell survival and stromal remodeling, creating a bidirectional metabolic support network (73). Metabolic interactions also occur among immune-cell subsets. Nutrient competition and metabolite exchange can create conditions in which immunosuppressive populations gain a selective advantage over effector lymphocytes (74). Altered autophagy, mitochondrial turnover, and lipid mobilization within immune cells can influence the local availability of amino acids and fatty acids, which may subsequently be utilized by TAMs, Tregs, or tumor cells. Such nutrient redistribution contributes to the establishment of metabolically distinct cellular niches within the pancreatic cancer microenvironment.

Amino acid metabolism constitutes an important component of this intercellular network. The tryptophan–kynurenine pathway, for example, generates immunoregulatory metabolites capable of activating AhR and suppressing antitumor immunity (75). Crosstalk among tumor cells, macrophages, and other stromal populations can therefore alter tryptophan availability and kynurenine production, reinforcing immune tolerance. Lipid mediators also participate in intercellular communication. Pro-inflammatory and pro-resolving lipid species can shape macrophage activation, lymphocyte function, and the balance between chronic inflammation and inflammatory resolution (76). Importantly, these metabolic interactions are spatially heterogeneous. Variations in oxygenation, nutrient availability, vascular perfusion, and stromal density generate distinct metabolic niches across different tumor regions (77). Immune cells residing in hypoxic tumor cores may therefore adopt metabolic and functional states that differ substantially from those located at the invasive margin or within relatively well-perfused stromal regions. This spatial metabolic heterogeneity influences immune-cell localization, infiltration depth, and functional competence (78). Collectively, intercellular metabolic crosstalk and niche-specific metabolic adaptation form an integrated metabolic symbiosis network that supports immune escape and pancreatic cancer progression (**Figure 2**).

**Figure 2:**
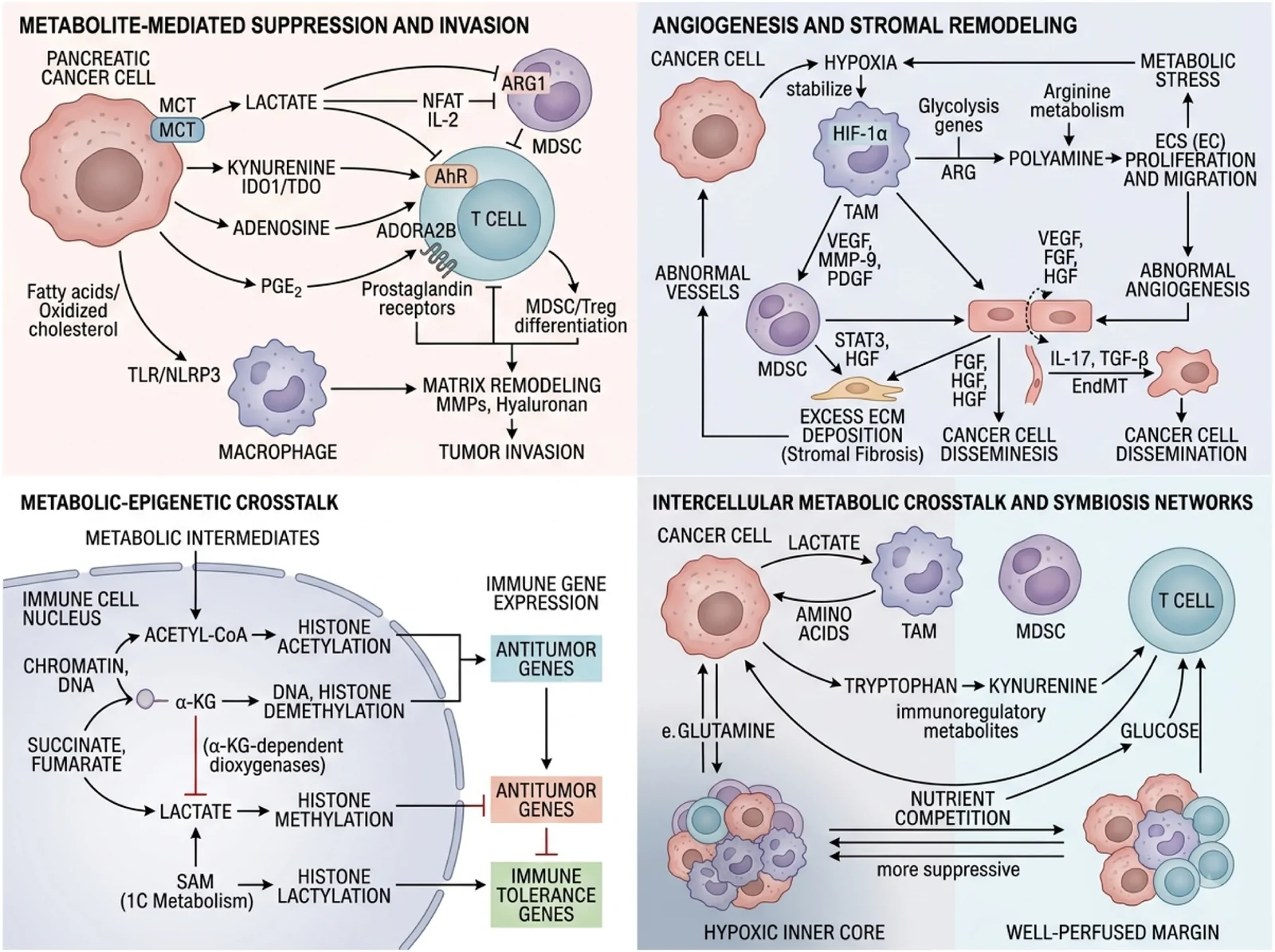
Mechanisms of immune cell metabolic reprogramming in pancreatic cancer progression.

## 5 Targeting immune cell metabolic reprogramming in pancreatic cancer

### 5.1 Metabolic enzyme inhibitors and pathway-targeted interventions

Targeting key metabolic enzymes represents an important strategy for reversing immune metabolic dysfunction in pancreatic cancer. Glycolytic enzymes such as lactate dehydrogenase (LDH) and pyruvate kinase M2 (PKM2) are frequently upregulated in pancreatic cancer and contribute to excessive lactate production and acidification of the TME. These metabolic alterations suppress the effector functions of T cells and NK cells (79). Pharmacological inhibition of LDH or PKM2 may reduce lactate accumulation and tumor glycolytic output, although excessive suppression of glycolysis may also constrain the metabolic activation of effector lymphocytes. Giving that lactate also regulates immune-cell function through epigenetic mechanisms such as protein lactylation, targeting glycolysis may simultaneously remodel both the metabolic and transcriptional landscape of the immune microenvironment (80).

Glutaminase (GLS) inhibition represents another potential therapeutic approach. Pancreatic cancer cells rely heavily on glutamine metabolism to sustain mitochondrial activity, redox homeostasis, and adaptation to hypoxia, whereas Tregs and TAMs can also exploit glutamine-dependent metabolic programs to maintain their immunosuppressive phenotypes (81). Targeting glutamine metabolism may restrict pancreatic cancer growth and immunosuppressive metabolic programs, but the same pathway also supports proliferating effector T cells, making dose, timing, and cellular selectivity critical determinants of therapeutic outcome. In addition, the LSD1–GLS2 axis has been implicated in regulating metabolic plasticity and chemotherapy sensitivity in pancreatic cancer, further supporting the therapeutic relevance of targeting glutamine metabolism (82).

FAO is also an attractive metabolic target because Tregs and MDSCs often depend on lipid oxidation to sustain survival and suppressive activity under nutrient-poor conditions. Pharmacological inhibition of FAO may reduce the persistence of these immunosuppressive populations and partially restore immune balance within the TME (83, 84). FAO inhibition may weaken lipid-adapted immunosuppressive populations, yet excessive suppression of mitochondrial lipid utilization could compromise memory T-cell persistence and long-term immune surveillance (85). Interestingly, pancreatic cancer cells can activate lipophagy-dependent FAO as an adaptive response following KRAS inhibition, suggesting that combining FAO inhibitors with oncogenic pathway-targeted therapies may overcome metabolic resistance (86).

Targeting amino acid-derived immunosuppressive pathways also has considerable therapeutic potential. The indoleamine 2, 3-dioxygenase (IDO)/tryptophan 2, 3-dioxygenase (TDO)–kynurenine pathway promotes immune escape by depleting tryptophan and generating kynurenine, which activates the AhR and promotes Treg differentiation while suppressing effector T-cell function. Blocking IDO or TDO may restore local tryptophan availability and reduce kynurenine-mediated immune suppression (87). The extracellular adenosine pathway represents another major metabolic checkpoint. CD73-mediated conversion of extracellular nucleotides promotes adenosine accumulation in pancreatic cancer, whereas adenosine receptor signaling inhibits T-cell and NK-cell proliferation, cytokine production, and cytotoxicity. Therapeutic inhibition of CD73 or blockade of adenosine receptors may therefore relieve this metabolically driven immunosuppressive circuit and enhance antitumor immunity (88).

### 5.2 Metabolic targeting and immunotherapy combination

Combining metabolic intervention with immune checkpoint blockade represents one of the most promising strategies for overcoming therapeutic resistance in pancreatic cancer. Effector T cells in pancreatic cancer are exposed to both inhibitory checkpoint signaling and severe metabolic stress, including glucose deprivation, lactate accumulation, hypoxia, and amino acid restriction. Therefore, simultaneously targeting immune checkpoints and metabolic suppressive pathways may restore T-cell function more effectively than either strategy alone (89). For example, inhibition of tumor-derived lactate production can improve the metabolic fitness of infiltrating lymphocytes and enhance sensitivity to immune checkpoint blockade. Targeting the ENSA lactylation–STAT3–CCL2 axis may similarly reduce immunosuppressive macrophage recruitment and improve responsiveness to checkpoint inhibition (90). Metabolic modulation may also enhance the immunological effects of radiotherapy and chemotherapy. Radiotherapy can induce immunogenic cell death and release damage-associated molecular patterns, thereby promoting innate and adaptive immune activation. When combined with strategies that improve immune-cell metabolism, these effects may be further amplified. For instance, local radiotherapy combined with CLDN18.2-targeted chimeric antigen receptor T (CAR-T) cells has been reported to enhance CAR-T-cell infiltration, increase pro-inflammatory macrophage polarization, reduce M2-like macrophages, and prolong survival in pancreatic cancer models (91). Proton radiotherapy combined with mesothelin-targeted CAR-T therapy has likewise shown synergistic activity by improving immune-cell trafficking and remodeling the TME (92).

Metabolic engineering of adoptive immune-cell therapies represents another emerging approach. CAR-T cells entering the pancreatic cancer microenvironment face severe metabolic constraints caused by hypoxia, glucose deprivation, high lactate concentrations, and lipid stress (93). Reprogramming CAR-T-cell metabolism before or during treatment may therefore improve their persistence and effector function. Strategies such as enhancing mitochondrial respiration, increasing metabolic flexibility, or supplying alternative metabolic substrates have shown promise in preclinical studies. For example, modulation of transcriptional regulators such as NR5A2 or metabolic conditioning with inosine may improve CAR-T-cell persistence and antitumor activity (94). Dual-targeting CAR-T platforms, such as FAP/CLDN18.2 CAR-T cells, may additionally reduce exhaustion and reshape the stromal microenvironment (95).

Nanotechnology-based delivery systems offer a further opportunity to improve the selectivity of metabolic interventions. Systemic administration of metabolic inhibitors can affect both tumor-promoting and antitumor immune populations, potentially limiting therapeutic efficacy. Nanocarriers can improve drug accumulation within tumors and may enable preferential delivery to selected immune-cell subsets, thereby widening the therapeutic window (96). Such platforms may be particularly useful for delivering inhibitors of lipid metabolism, amino acid metabolism, or post-translational metabolic modifications. Importantly, the optimal combination strategy is likely to vary among patients because pancreatic cancer exhibits substantial intertumoral metabolic heterogeneity. Differences in glycolysis, mitochondrial metabolism, lipid utilization, stromal composition, and immune-cell infiltration may influence therapeutic sensitivity (97). Integrating metabolic profiling with immune characterization may therefore enable biomarker-guided selection of rational combination therapies.

### 5.3 Metabolic targets and immunometabolic editing technologies

Recent discoveries in metabolite-driven post-translational modifications have expanded the landscape of potential immunometabolic targets. Protein lactylation, particularly histone lactylation, has emerged as a key mechanism linking altered glycolysis to transcriptional regulation. In pancreatic cancer, elevated histone H3 lysine 18 lactylation (H3K18la) has been associated with aggressive biological behavior and poor prognosis (98). A glycolysis–H3K18la–TTK/BUB1B positive-feedback loop has been implicated in sustaining malignant progression, suggesting that lactylation-associated pathways may represent therapeutic vulnerabilities. Lactylation may also influence stromal remodeling and metastatic behavior through stabilization or modification of proteins such as S100A11 and ANXA2, further connecting tumor metabolism with the immune and stromal microenvironment (99, 100). Advances in single-cell and spatial metabolic technologies are providing unprecedented opportunities to resolve metabolic heterogeneity within pancreatic tumors. Single-cell transcriptomics, spatial transcriptomics, imaging mass spectrometry, and emerging single-cell metabolomic platforms can identify metabolically distinct tumor, stromal, and immune-cell subsets and define their spatial organization (101). Such approaches are particularly valuable in pancreatic cancer because metabolic programs differ substantially between hypoxic tumor cores, invasive margins, fibrotic stromal regions, and vascular niches. Spatial metabolic profiling has already begun to reveal how glycolytic CAF populations and lactate-rich niches contribute to tumor progression and neural invasion (102).

Genome-editing technologies provide another strategy for directly modifying immune-cell metabolic programs. clustered regularly interspaced short palindromic repeats (CRISPR)-based editing can be used to disrupt exhaustion-associated genes or activate metabolic pathways that enhance immune-cell fitness (103). In CAR-T cells, deletion of transcriptional regulators such as ID3 and SOX4 has been reported to improve antitumor activity in pancreatic cancer models (104). *In vivo* CRISPR screening can also identify tumor-intrinsic pathways that regulate susceptibility to immune attack, including metabolic and oxidative-stress programs such as NRF2 signaling (105). These approaches may facilitate the development of metabolically optimized cell therapies and identify biomarkers of therapeutic response. Mitochondrial manipulation represents a particularly novel frontier (106). Because chronic metabolic stress in the pancreatic cancer microenvironment can impair mitochondrial biogenesis, OXPHOS, and mitochondrial quality control in T cells, strategies aimed at restoring mitochondrial fitness may reverse immune exhaustion. These approaches include pharmacological enhancement of mitochondrial function, modulation of mitochondrial dynamics, and experimental mitochondrial transfer (107). Although most of these strategies remain at an early preclinical stage, they provide a conceptual framework for directly restoring the metabolic competence of dysfunctional antitumor immune cells. Despite these advances, immune-metabolic therapy in pancreatic cancer remains challenged by substantial pathway redundancy and compensatory adaptation. Blocking a single metabolic route may trigger alternative nutrient utilization pathways in both tumor and immune cells (108). Future therapeutic strategies will therefore likely require context-specific combinations that simultaneously target tumor metabolism, immunosuppressive immune-cell programs, and stromal metabolic support.

## 6 Future directions

Despite rapid advances, several fundamental questions remain unresolved in pancreatic cancer immunometabolism. A central challenge is identifying metabolic dependencies that can be selectively exploited in immunosuppressive populations while preserving the fitness of cytotoxic lymphocytes. Many pathways, including FAO, glutamine metabolism, mitochondrial respiration, and redox control, are shared across multiple immune populations, making therapeutic selectivity a major barrier to translation (109). Resolving this problem will require a more precise understanding of how metabolic programs are configured across distinct immune states and how these programs interact with tumor and stromal metabolism. Another major question is whether immune-metabolic states differ between primary pancreatic cancer and metastatic lesions. Liver, lung, peritoneal, and lymph-node metastases expose immune cells to distinct nutrient landscapes, oxygen tensions, stromal architectures, and metabolite pools, potentially creating site-specific metabolic programs (110). Defining these differences may reveal metastatic-site-specific vulnerabilities that are not apparent from primary-tumor profiling alone.

Treatment itself is likely to reshape the immune-metabolic landscape. Cytotoxic chemotherapy alters nutrient availability, redox stress, mitochondrial function, and cell-death-associated metabolite release, yet the resulting effects on T cells, macrophages, MDSCs, NK cells, and fibroblast–immune metabolic exchange remain incompletely characterized. Longitudinal profiling before, during, and after therapy will therefore be critical for understanding how immune metabolism evolves under therapeutic pressure and for distinguishing transient metabolic adaptation from stable reprogramming (111). Temporal dynamics represent an equally important dimension. Immune-cell metabolic phenotypes are unlikely to remain static during tumor progression. Early inflammatory states may evolve into metabolically adapted immunosuppressive programs, while treatment-resistant tumors may establish new nutrient-sharing and redox networks (112). Integrating serial tissue sampling with single-cell, spatial, and metabolomic approaches may reveal how metabolic states transition over time and identify windows in which metabolic intervention is most effective. Finally, clinical translation will depend on robust biomarkers capable of identifying patients most likely to benefit from immunometabolic therapy. Candidate biomarkers may include circulating metabolites, metabolic enzyme expression, spatial metabolite distributions, immune-cell metabolic signatures, and composite multi-omic profiles (113). The most informative biomarkers will likely integrate metabolic state with immune composition, stromal architecture, and treatment history rather than rely on a single pathway. Establishing such biomarkers will be essential for moving immunometabolic therapy from broadly applied pathway inhibition toward biomarker-guided precision treatment in pancreatic cancer (114).

## 7 Conclusion

Immune cell metabolic reprogramming has emerged as a central mechanism linking the unique metabolic landscape of pancreatic cancer to the establishment and maintenance of an immunosuppressive TME. Rather than representing a passive adaptation to nutrient deprivation and hypoxia, metabolic remodeling actively determines immune-cell differentiation, functional polarization, persistence, and exhaustion. In pancreatic cancer, impaired glucose and amino acid availability, excessive lactate accumulation, altered lipid metabolism, and dysregulated mitochondrial function collectively suppress the activity of CD8^+^ T cells and NK cells, while favoring the survival and immunosuppressive functions of Tregs, TAMs, and MDSCs. Moreover, metabolites such as lactate, kynurenine, succinate, and lipid-derived mediators can function as signaling molecules and epigenetic regulators, thereby establishing an integrated metabolism–immunity–epigenetics network that reinforces immune escape and tumor progression.

An important challenge in this field is that metabolic dependencies are shared across multiple immune-cell populations. For example, inhibition of glutamine metabolism may suppress MDSCs and tumor-supportive macrophages but may also impair the proliferation of activated T cells, whereas blockade of FAO may reduce Treg-mediated immunosuppression while potentially compromising the long-term persistence of memory T cells. Therefore, nonspecific inhibition of individual metabolic pathways is unlikely to provide optimal therapeutic benefit. Future strategies should instead focus on cell type-specific and context-dependent metabolic interventions, including targeted nanoparticle delivery, metabolic engineering of adoptive T cells, and selective modulation of immune-cell metabolic checkpoints. Such approaches may allow suppression of tumor-promoting metabolic programs while preserving or enhancing antitumor immunity.

Combining metabolic targeting with immunotherapy is particularly promising for overcoming the intrinsic resistance of pancreatic cancer to immune checkpoint blockade. Metabolic interventions that reduce lactate accumulation, restore amino acid availability, disrupt adenosine or kynurenine signaling, or improve mitochondrial fitness may enhance T-cell infiltration and effector function and thereby increase responsiveness to PD-1/PD-L1 or CTLA-4 blockade. Similarly, metabolic optimization of CAR-T cells and other adoptive immune-cell therapies may improve their persistence and functionality within the hypoxic, nutrient-poor, and highly fibrotic pancreatic cancer microenvironment. Nevertheless, the systemic consequences of metabolic manipulation, including effects on normal tissues and endocrine homeostasis, must be carefully evaluated during clinical translation.

Future research could place greater emphasis on resolving the temporal and spatial heterogeneity of immune metabolism in pancreatic cancer. The integration of single-cell and spatial transcriptomics, metabolomics, proteomics, and epigenomics will enable precise mapping of metabolically distinct immune-cell states and their interactions with tumor and stromal compartments. In parallel, patient-derived organoids, humanized mouse models, and ex vivo tumor–immune co-culture systems will be important for validating immune-metabolic vulnerabilities in clinically relevant settings. Development of dynamic metabolic biomarkers and noninvasive imaging approaches may further facilitate patient stratification and real-time assessment of therapeutic response. Overall, the future of pancreatic cancer immunometabolism lies in integrating mechanistic understanding, cell-specific metabolic targeting, and biomarker-guided combination therapy. By selectively restoring metabolic fitness in antitumor immune cells while disrupting the metabolic adaptations that support immunosuppressive populations, it may be possible to convert pancreatic cancer from an immunologically “cold” tumor into a more immune-responsive state. Such strategies hold considerable potential to overcome current therapeutic resistance and provide new opportunities for durable clinical benefit in patients with pancreatic cancer.
